# Match-play running activity in elite female soccer field and assistant referees

**DOI:** 10.5114/biolsport.2025.147014

**Published:** 2025-03-24

**Authors:** Christopher Carling, Franck Doudet, Batiste Gruson, Jean-Michel Prat, Thomas Pavillon

**Affiliations:** 1 FFF Research Centre, French Football Federation, Clairefontaine National Football Centre, Clairefontaineen-Yvelines, France; 2 Laboratory Sport, Expertise and Performance (EA 7370), French Institute of Sport (INSEP), Paris, France; 3 French National Referees Association, French Football Federation, Clairefontaine National Football Centre, Clairefontaine-en-Yvelines, France

**Keywords:** Match officials, External loads, Competition, Fatigue, Sprinting

## Abstract

This study aimed to 1) describe running outputs during official match-play in elite French female Field (FR) and Assistant Referees (AR), 2) determine whether decrements in outputs occurred in the 2^nd^-half and during the final 15-minute interval of each half. A total of 13 elite female game officials participated: 6 AR (age: 28.9 ± 5.1) and 7 FR (age: 28.1 ± 2.0). Outputs were collected using GPS over 3 consecutive seasons (2020/21–2022/23). Competitions included elite senior female/male domestic matches and international female/male youth & senior matches (n = 501; AR = 285, FR = 216). Variables included: total distance covered (km), distances travelled (m) at low- (< 13 km/h) and high-speeds (≥ 13 km/h, ≥ 19 km/h, ≥ 23 km/h) and the frequency of high-intensity acceleration & deceleration events (≥ 3 m · s^−2^). Mean outputs per minute were compared across match halves and for the first 15-minute versus the final 15-minute interval in each half using Effect Sizes (ES). Main results showed that FR covered approximately 9.5 km per game of which 24% was at high-speeds (D ≥ 13 km/h) while AR travelled ~5 km (~14% ran at high-speeds). More high-intensity deceleration versus acceleration events were observed for both roles. Outputs generally dropped in the 2^nd^ versus the 1^st^ half (FR = small to moderate and AR = small to large ES) and during the final 15-minute intervals in each half versus the first 15-minutes in the 1^st^-half (FR = moderate to large and AR = small to large ES). This study has established general match running activity profiles for elite French assistant and field referees and reported a trend for a temporal decline in outputs whilst demonstrating the importance of performing deceleration events.

## INTRODUCTION

Sports officials play a crucial role in overseeing and adjudicating organized sport and competition. While their contribution is evidently substantial, scientific evidence to inform their specialized training at various levels has arguably lagged [1] and notably in comparison to the athletes they officiate. In soccer, and particularly at elite standards of play, science applied to refereeing is still in its infancy despite an increase in the number of scientific investigations over the last two decades [2]. Research has nevertheless provided pertinent information regarding officials’ anthropometric and fitness profiles and physical outputs (e.g., external and internal loads) in competition [3]. However, most of the research conducted in elite match officiation settings has focused on male referees. Expanding this to include female officials [4] is necessary especially when considering the heightened physical demands observed in the contemporary elite female game [5] and the rise in the number of female referees officiating in male football competitions [2] where they potentially face an increase in physical demands. Indeed, recent research [6] comparing demands in elite male and female referees in their respective competitions, reported significantly greater differences in the total distance covered and the frequency of sprints and high-intensity acceleration and deceleration events performed by the former. Similarly, researchers have typically focused upon field referees although some information regarding physical outputs in assistant officials is available [7–10]. These studies conducted in male assistant officials have identified substantial differences in the physical outputs generated compared with field peers. To our knowledge, only one study [11] has reported data on competitive physical demands in female assistant officials and this included a small sample size of matches (n = 10).

In general, irrespective of gender or role, referees´ movements during match-play are characterized by low-intensity activities such as walking and jogging. These are intermittently interspersed with running actions performed at high-intensities [6–12]. Female field and assistant referees are shown to cover total distances approaching 10 km [6, 13, 14] and approximately 5.5 km [11] per match respectively. High-intensity metrics previously reported as a percentage of the total distance covered, equated to 29% (distances > 12 km/h) [13] and 36% (distance > 13 km/h) [11] respectively. Although the current literature has arguably helped build a more objective framework to characterise game demands and can subsequently help prescribe physical conditioning in elite female referee populations, data is currently only available from a small number of studies [6, 11, 13, 14]. As such, research is warranted to generate additional profiles and compare these with data from other elite referee cohorts and the activity profiles of elite outfield players, as well as expanding the current body of literature. Similarly, information specifically relating to temporal deteriorations in running outputs and the potential occurrence of physical fatigue in female officials is to our knowledge very limited. In elite field officials [13, 14] significant differences in the total distance run across match halves was observed while this was not the case in elite assistant referees [11]. Regarding high-intensity activity in these three studies, no significant change was observed across halves for either role. However, of these three studies, only one examined change in the frequency of high-intensity acceleration and deceleration events across halves (significant decrease observed) [13] and two analysed running activity across 15-minute periods with contrasting findings reported for high-intensity outputs in field [14] and assistant [11] officials using data derived from the same sample of matches. As such, additional studies are warranted to confirm or refute these findings and strengthen the current limited evidence base whilst providing information for designing conditioning strategies to offset potential declines in match performance owing to fatigue. This study aimed to 1) describe physical outputs using measures of external load in elite French female CR and AR during official match-play, 2) determine whether decrements in outputs occurred across match halves and/or during the final 15-minute intervals of each match half.

## MATERIALS AND METHODS

### Participants & match sample

A total of 13 elite female match officials registered to the French National Football Referees Association participated. The cohort included 6 elite Assistant Referees (AR, age 28.9 ± 5.1) and 7 elite French Field Referees (FR, age 28.1 ± 2.0). Data were collected over 3 consecutive seasons (2020/21–2022/23). The sample of matches included female and male French domestic national league (female D1 and D2 and male D4) and female national cup and international youth & senior matches. It comprised a total of 501 official matches of which 285 were completed by AR (range: 27–73 matches) and 216 by FR (range: 16–53 matches). Only 90-minute matches in which the officials completed the entire duration of the match were included. The sample did not include any matches where extra-time occurred. Data were analysed for the entire match and across match halves and 15-minute intervals. Any added time for stop-pages was included for each half and the final 15-minute period of each half.

At the beginning of each competitive season, the officials received comprehensive verbal and written explanations of the GPS monitoring process in place. This monitoring was a condition of the French Football Federation female football referees’ development programme, part of which their match performances were routinely measured over the course of the competitive season [15]. Nevertheless, the officials gave their written informed consent to participate in the monitoring and all associated research programmes for which there was secondary usage of the data collected. Permission for the study was also obtained from the French National Football Referees Association (FNFRA). To ensure confidentiality, all performance-related data were fully anonymized prior to analysis.

### Procedures

All officials wore a portable GPS tracking device (Catapult One, Catapult, Australia), sampling at 10 Hz. The GPS system was placed in a customized vest and worn between the scapulae. To limit potential inter-unit variability, each official wore the same unit for the entire duration of the present study. Twenty minutes prior to the start of each match, each official activated their GPS unit to ensure clear satellite reception. Information regarding the number of satellites and horizontal dilution of precision was unavailable. These devices have undergone Fédération Internationale de Football Association (FIFA) Electronic Player Tracking Systems quality certification (https://www.fifa.com/technical/football-technology/resource-hub?id=a6672a86658847eb8b4131b56c4de875).

Prior to the start of each match, the game officials performed a standardized 15-minute warm-up including running, progressive sprints and stretching. This data was excluded from the final analysis. External load variables analysed during match-play included: total distance covered (TD), total distance covered at low speeds < 13 km/h (D < 13 km/h) and high speeds ≥ 13 km/h (D ≥ 13 km/h), ≥ 19 km/h (D ≥ 19 km/h), and ≥ /h (D > 23 km/h). The frequency of high-intensity acceleration & deceleration events (≥ 3 m · s^−2^) was also analysed. Relative values for these variables are also reported (distances covered per minute of play). The present speed thresholds were selected at the start of the study by the FNFRA to enable comparisons with data observed on match running activity in contemporary elite female outfield football players [5, 16, 17] as activity profiles in football officials are reported to be associated with those observed in outfield players [18, 19]. Also, in other elite female officials, movements performed at speeds greater than 13 km/h were classed as high-intensity activities [11, 14]. Finally, the highest values observed for maximal speed attained (km/h) across the dataset were reported.

### Statistical analysis

Results are presented as mean ± standard deviations for the overall values obtained for each performance variable. These calculations were performed using Excel (Microsoft, Richmond, USA). Percentage changes in the variables concerning distances run were determined to compare running activity across match halves and for the first 15-minute match interval versus the final 15-minute interval in each half. Running outputs for the different match intervals were analysed as the distances covered per minute of play to account for any added time included at the end of each half and which might bias the interpretation of results (18). Percentage changes in mean values across halves and the selected 15-minute periods in each half were calculated with 90% confidence limits (90% CL) using a specifically designed Excel spreadsheet [20]. A magnitude-based decision [21] approach to statistical comparisons was adopted using Cohen’s Effect sizes (ES). ES and associated 90% CL were quantified to indicate the practical meaningfulness of the differences in mean values across match periods. Differences were classified as *trivial* (< 0.2), *small* (> 0.2–0.6), *moderate* (> 0.6–1.2), *large* (> 1.2–2.0) and *very large* (> 2.0–4.0).

## RESULTS

### Overall match-play running outputs

Table 1 presents the overall match running demands observed in field and assistant officials. On average, field referees covered a TD of 9481.0 ± 947.8 m per match. Of this 24.0% was covered at speeds ≥ 13 km/h (2277.6 ± 562.9 m), 4.2% ≥ 19 km/h, (399.0 ± 149.7 m) and 0.5% ≥ 23 km/h (48.7 ± 37.4 m). These distances equated to 75.9 m covered per minute overall, 24.3 m at > 13 km/h and 4.2 m at ≥ 19 km/h. The maximum recorded distance for overall TD and those covered at ≥ 13 km/h and ≥ 19 km/h, were 11696.5 m, 3618.3 m and 796.5 m respectively. Additionally, the mean frequency of high-intensity acceleration events per match was 34.4 (0.4/min), with a maximum of 139, while deceleration events had a mean of 45.3 (0.5/min) and a maximum of 127.

**TABLE 1:** Distances covered at different speed thresholds and frequency of high-intensity acceleration and deceleration events in field and assistant female game officials.

Match role	Field Referees				Assistant Referees			

Variable	Mean (m)	Mean m/minute	Maximum (m)	Maximum m/minute	Mean (m)	Mean m/minute	Maximum (m)	Maximum m/minute
Overall	9481.0 ± 947.8	100.3 ± 9.8	11696.5	124.0	5052.5 ± 734.9	53.1 ± 7.6	6589.9	67.61

< 13 km/h	7203.4 ± 568.4	75.9 ± 6.1	8563.5	91.3	4353.9 ± 501.8	45.7 ± 5.2	5414.1	57.5

> 13 km/h	2277.6 ± 562.9	24.3 ± 5.8	3618.3	38.2	698.6 ± 322.7	7.3 ± 3.4	1776.6	18.0

> 19 km/h	399.0 ± 149.7	4.2 ± 1.6	796.5	8.2	96.3 ± 78.4	1.0 ± 0.8	379.3	3.9

> 23 km/h	48.7 ± 37.4	0.5 ± 0.4	211.5	2.2	7.7 ± 13.4	0.1 ± 0.1	92.1	1.0

**Variable**	**Mean N°**	**Mean N°/minute**	**Maximum n°**	**Maximum N°/minute**	**Mean N°**	**Mean N°/minute**	**Maximum n°**	**Maximum N°/minute**

Accelerations > 3 m · s^−2^	34.4 ± 24.2	0.4 ± 0.3	139	1.5	34.0 ± 16.1	0.4 ± 0.2	73	0.82

Decelerations > 3 m · s^−2^	45.3 ± 21.6	0.5 ± 0.2	127	1.3	49.7 ± 21.3	0.5 ± 0.3	83	0.93

For ARs, the mean TD per match was 5052.5 ± 734.9 m, of which 13.8% (698.6 ± 322.7 m) was covered at speeds ≥ 13 km/h, 1.9% (96.3 ± 78.4 m) at ≥ 19 km/h, and 0.2% (7.7 ± 13.4 m) at ≥ 23 km/h. The maximum distances for TD, ≥ 13 km/h, and ≥ 19 km/h were 6589.9 m, 1776.6 m, and 379.3 m, respectively. The mean and maximum frequencies for high-intensity acceleration and deceleration events were 34.0 ± 16.1 (0.4/min) and 73, and 49.7 ± 21.3 (0.5/min) and 83, respectively.

The maximal speeds attained across the entire sample of FRs and ARs were 31.4 km/h and 28.7 km/h, respectively.

### In-match changes in running outputs

For FRs, mean values for all variables decreased in the second half (Table 2). Changes ranged from -5.8% [-5.1, 6.5] for distance covered at speeds < 13 km/h to -22.4% [-19.1, -25.5] for the frequency of high-intensity deceleration events per minute. Effect size (ES) differences for the half-to-half changes ranged from a small effect size of -0.3 [-0.24, -0.37] for the decrease in high-intensity acceleration events, to a moderate effect size of -0.8 [-0.72, -0.92] for overall distance covered.

**TABLE 2:** Distances covered per minute at different speed thresholds and frequency of high-intensity acceleration and deceleration events in field and assistant female game officials across match halves.

Match role	Field Referees				Assistant Referees			

	Mean m/minute				Mean m/minute			

Variable	1^st^-half	2^nd^-half	% Change (90% CI)	ES (90% CI)	1^st^-half	2^nd^-half	% Change (90% CI)	ES(90% CI)
Overall	103.3 ± 10.9	96.4 ± 9.8	-6.6 (-5.9, -7.3)	-0.74 (-0.66, -0.83)	54.5 ± 9.0	51.9 ± 9.5	-5.2 (-3.1, 7.2)	-0.52 (-0.31, -0.74)

< 13 km/h	78.2 ± 6.8	73.6 ± 6.3	-5.8 (-5.1, -6.5)	-0.82 (-0.72, -0.92)	47.2 ± 6.9	44.5 ± 6.9	-5.7 (-3.7, 7.7)	-1.26 (-0.82, -1.70)

> 13 km/h	25.1 ± 6.4	22.8 ± 6.1	-9.7 (-7.6, -11.8)	-0.43 (-0.34, -0.53)	7.3 ± 3.6	7.3 ± 4.0	0.0	-0.01 (-0.003,0.11)

> 19 km/h	4.4 ± 1.7	3.9 ± 1.9	-15.6 (-10.5, -20.3)	-0.40 (-0.24, -0.55)	-	-	-	-

**Variable**	**Mean N°/minute**				**Mean N°/minute**			

Accelerations > 3 m · s^−2^	0.4 ± 0.3	0.3 ± 0.2	-21.7 (17.7, 25.6)	-0.30 (-0.24, -0.37)	0.4 ± 0.2	0.3 ± 0.2	-16.4 (-10.6, -21.9)	-0.35 (-0.25, -0.45)

Decelerations > 3 m · s^−2^	0.5 ± 0.3	0.4 ± 0.2	-22.4 (19.1, 25.5)	-0.51 (-0.43, -0.58)	0.6 ± 0.3	0.5 ± 0.3	-19.6 (-14.6, 24.3)	-0.44 (-0.34, -0.53)

Similarly, outputs for all variables decreased during the second half in ARs, except for distance covered at speeds ≥ 13 km/h, where the half-to-half values were almost identical. The range of changes in ARs varied from -5.2% [-3.1, -7.2] for TD to a maximum of -19.6% [-14.6, -24.3] for the frequency of high-intensity deceleration events. The ES for these changes ranged from a small effect size of -0.4 for high-intensity acceleration events to a large effect size of -1.3 for distance covered at speeds < 13 km/h.

**FIG. 1:**
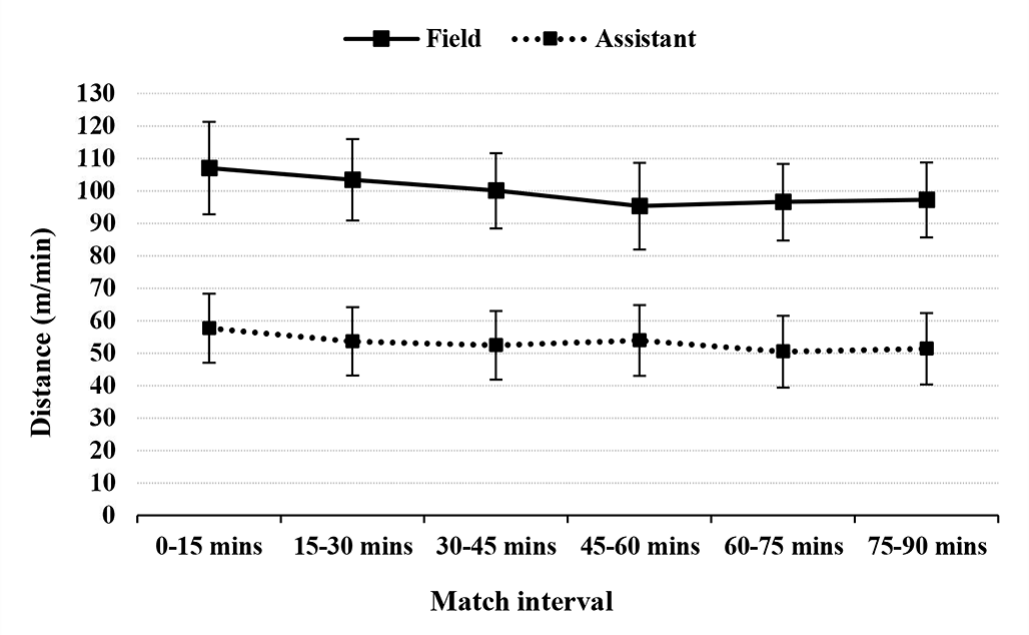
Overall distance covered (in metres/minute) across 15-minute match intervals in elite female field and assistant referees.

Figures 1 through to 4 illustrate running performance (TD and distance covered at speeds > 13 km/h) across 15-min match intervals for both FRs and ARs. Due to low occurrences, comparisons of distances covered at speeds ≥ 19 km/h and ≥ 23 km/h were not possible.

**FIG. 2:**
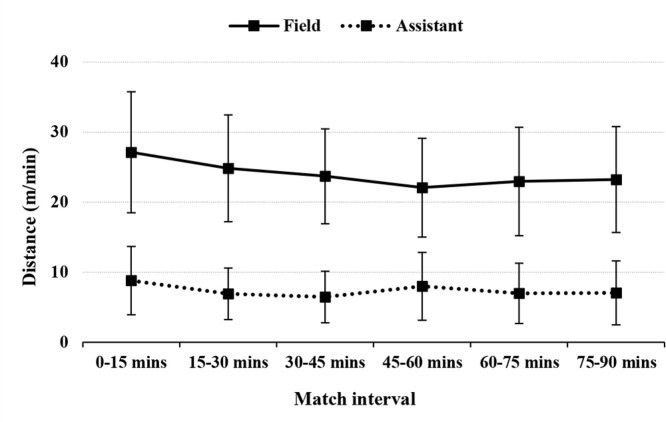
Distance covered at ≥ 13 km/h (in metres/minute) across 15-minute match intervals in elite female field and assistant referees.

For FRs, the respective changes in TD and distance covered at > 13 km/h between the first and last 15-min match intervals in the first half were:

– 107.0 ± 14.2 m/min vs 100.0 ± 11.6 m/min (-6.3% [-5.1, 7.5], ES = -0.69 [-0.56, -0.82], moderate)
– 27.1 ± 8.7 m/min vs 23.7 ± 6.8 m/min (-11.6% [-8.4, -14.7], ES = -0.62 [-0.46, -0.78], moderate).

In the second half, the final 15-min interval compared to the first 15-minute interval showed:

– 107.0 ± 14.2 m/min vs 97.2 ± 11.5 m/min (-9.0% [-7.8, -10.2], ES = -0.99 [-0.86, -1.12], large),
– 27.1 ± 8.7 m/min vs 23.2 ± 7.6 m/min (-14.7% [-11.3, -18.1], ES = -0.69 [-0.53, -0.85], moderate).

For ARs, TD and distance covered at ≥ 13 km/h were lower in the first 15-min interval compared to the last 15-min interval in the first half:

– 57.2 ± 10.6 m/min vs 52.4 ± 10.6 m/min (-9.4% [-7.6, -11.2], ES = -0.69 [-0.55, -0.82], moderate),
– 8.8 ± 4.9 m/min vs 6.5 ± 3.7 m/min (-31.4% [-25.7, -37.6], ES = -0.70 [-0.59, -0.82], moderate).

In the second half, the final 15-min interval compared to the first 15-min interval showed:

– 57.2 ± 10.6 m/min vs 51.3 ± 11.0 m/min (-11.6% [-9.2, -13.9], ES = -1.19 [-0.94, -1.43], large),
– 8.8 ± 4.9 m/min vs 7.0 ± 4.6 m/min (-26.9% [-20.2, -33.1], ES = -0.53 [-0.39, -0.67], small).

## DISCUSSION

In this study in elite French female field (FR) and assistant (AR) soccer officials, running outputs derived from GPS measures of external load in competition and potential changes in performance over the course of play were determined. Main findings were that FR ran approximately 9.5 km per game of which 24% was covered in high-speed activities (≥ 13 km/h) while AR travelled ~5 km (~14% performed at high-speed). Both FR and AR reported more high-intensity deceleration than acceleration events. In FR and AR respectively, analysis of 2^nd^ versus 1^st^ half outputs, showed small to moderate and small to large reductions while moderate to large and small to large decreases were respectively observed during the final 15-minute intervals of each half versus the first 15-minute interval of play.

### Overall match-play running outputs

The limited data currently available for elite female field referees has reported a mean total distance of 9946 m and 308 m covered in high-intensity movement (> 15.1 km/h) in Spanish officials [6] and 10032 m and 3137 m (> 13 km/h) in peers officiating in U20 international matches [14]. Here, officials travelled on average, lower total distances (9481 m). Potential explanations for discrepancies in findings across studies include the data collection methods employed and/or contextual and cultural differences related to the demands (e.g., tactical, physical intensity) specific to the competitions in which they typically officiate [22–24]. However, of note, is the maximum total reported distance of ~11700 m and 5 out of the 7 present officials reported distances over 11 km and on at least one occasion, all 7 covered over 10.5 km in any one match. These values demonstrate the extreme overall demands that are inherent to contemporary elite French female soccer refereeing. Regarding high-speed activities, 24.0% of the total distance was covered at speeds ≥ 13 km/h and 4.2% at ≥ 19 km/h. In the only study [14] to our knowledge that has employed a similar speed threshold for high-speed running (> 13 km/h), a value of ~31% was reported. Interestingly, comparisons with the proportion of the total distance covered at very high running speeds (> 19 km/h) by female outfield players participating in the 2023 FIFA World Cup [17] and 2022 UEFA European Championships [5], reported not too dissimilar values to those observed in the present officials (4.2% here versus respectively 6.5% and 6.2%).

Regarding assistant referees, these covered a mean total distance of 5052 m of which ~14% (~699 m) was covered at speeds ≥ 13 km/h and 1.9% (~96 m) ≥ 19 km/h. Comparative research conducted in 14 top-class assistant referees [11], albeit using a different methodology and a substantially smaller sample of 10 matches, reported considerably greater values: 5594 m and 1999 m (36% of the total) for total distance covered and that run at high-speeds (> 13 km/h). Again, of interest here in the present cohort are the maximum values of ~6590 m and ~1777 m while all 7 assistants covered a total distance of over 6 km on at least one occasion. This information demonstrates a need to prepare officials to respond to the intense demands of play that are sporadically and substantially greater than those they are typically accustomed to performing. Research to identify the contexts in which these greater running outputs occur is arguably warranted. For example, research has shown that the physical efforts performed by male referees are related to the ranking of the teams they are officiating [24] as well as the nature of the competition [7] (domestic versus European matches for example).

Here, both FR and AR reported a greater number of high-intensity deceleration than acceleration events. This result is in line with recent data generally reported in elite male and female referees [18] and in two reviews of elite female [25] and male [26] outfield soccer players. Training strategies for developing deceleration capacities should look to enhance referees’ ability to skillfully dissipate braking loads, develop mechanically robust musculoskeletal structures, and ensure frequent high-intensity horizontal deceleration exposure (27). Interestingly, AR and FR performed a similar number of acceleration and deceleration actions. This result implies a need to adopt a generic approach to conditioning programmes to develop these two aspects of locomotor performance in elite female referee populations.

Finally, regarding running speed, maximal values of 31.4 km/h and 28.7 km/h were reported in CR and AR respectively. Research in Spanish female field referees has also observed speeds sporadically approaching 30 km/h [6] while the present values are similar to those observed in international female outfield players [5]. Combined with the above data on deceleration and acceleration events, these findings have important practical implications for physical conditioning programmes to ensure that elite female officials are optimally prepared for the high-intensity mechanical loading demands of competition.

### In-match changes in running outputs

A substantial number of articles [7–10, 18, 19, 28–32] have investigated alterations in running outputs in elite male match officials over the course of match-play. This body of work contrasts with the limited information currently published on female peers [11, 13, 14] which has nevertheless reported a trend for reductions in running activity and notably the total distance covered across match halves. Here, a decrease in outputs across halves was generally observed in both roles (effect sizes range: trivial to large) with the largest effect size differences observed for total distance run and distance travelled in low-speed running. The reasons for these decreases may be multifactorial (e.g., physical and mental fatigue). For example, the marked reduction in exercise activity in female soccer players has been related to substantial reductions in muscle glycogen concentration although similar research in female is necessary in referees to verify this finding [33]. Contextual factors (e.g., standard of play, team ranking, match type, style of play, tempo, interruptions, pacing strategies) can also be linked to the variations observed in movement patterns in officials over the course of play [10, 28, 30, 31]. However, to our knowledge, no studies have investigated the potential effects of these contextual factors on locomotor activity in female officials. Determining whether contextual factors impact the declines in physical outputs in assistant and field referees differently is also of interest. Do certain teams’ style of play for example have a greater impact on the linear movements typically observed in assistant referees?

Interestingly, the highest values observed for the percentage decreases in running metrics in the second half concerned the frequency of high-intensity acceleration and deceleration events respectively (-16.4% and -22.4%, small ES). In the only other study [13] to our knowledge that has analysed these metrics in game officials, similar decreases were observed (-17.8% and -17.6%, small ES) while comparable data has also been reported in high-level outfield soccer players [26]. These results suggest that akin to observations on high-intensity acceleration and deceleration actions in players, game officials are subject to neuromuscular and mechanical fatigue (e.g., represented by accumulation of tissue damage, loss of stiffness and strength qualities) [26]. This fatigue may have a particularly profound effect on changes to match-related movement ability and efficiency and is of significant interest to physical conditioning coaches.

Previous research has shown that reductions in running activity occurring in the final stages of play are similar in female assistant [11] and field [14] referees. However, these were consistently able to maintain their distance in relation to the offside line and position of the ball respectively. These results could suggest that officials adopt individual pacing strategies to control their efforts and conserve energy for critical moments towards the end of play. In the present cohort, decreases (*small, moderate* and *large effect size differences*) in the total distance covered and in that ≥ 13 km/h occurred in the final 15-minute interval of each half versus the first 15-minute interval in both field and assistants. These findings would suggest the development of fatigue and an inability to maintain physical outputs. However, running activity is typically at its highest during the first 15-minutes of play in match officials (9) and players (34) and officials are shown to align their efforts with those observed in the elite players’ they referee [35] which also tend to decline over the final third of play (34). This might also have been the case in the present cohort partly explaining the decline in running outputs. It is note-worthy however that values for total and high-speed distance were not always at their lowest during the final 15-minutes of play in comparison with other 15-minute intervals, again suggesting a pacing strategy. Additional research in other elite female referee populations and factoring in contextual factors is warranted to verify these findings. Research into the potential link between fitness measures and changes in movement patterns over the course of play would also be pertinent. A recent investigation [13] has shown that overall running outputs in a cohort of elite Spanish female referees were statistically associated to their performances in tests of repeated sprint ability and intermittent recovery (YYIR1 test). However, no information exists as to whether greater fitness capacities would enable female referees to maintain running outputs over the course of matches and help them to keep up with play.

### Limitations

Although the current match sample size can be considered substantial (> 400 matches over a 3-season period), data were derived from a relatively small cohort of elite female officials (n = 13). From 2024 onwards, an additional 7 officials will integrate the current elite monitoring programme enabling the French National Referees Association to progressively obtain a larger picture of the movement patterns in its elite female refereeing population. Similarly, the potential impact of key contextual factors such as playing standard on running outputs could not be investigated as only a small proportion of the matches were played at international standards or at different levels in the domestic game. In addition, it was not possible to extract data from the GPS manufacturer’s software regarding short rolling periods of peak running activity (e.g., sprint activity over a 5-minute period). This would have provided pertinent data relating to the extreme demands experienced over the course of play and how outputs evolved over the ensuing period. Such data can notably indicate the officials’ ability to recover following intense periods of activity [31, 32] and identify the running volumes and intensities necessary for designing conditioning drills based upon the most-demanding periods [36]. Finally, information on internal strain (e.g., ratings of perceived exertion, heart-rate) [37] would have complemented the external load data.

## CONCLUSIONS

In general, there is a lack of research investigating performance-related demands in elite female sports officials and in soccer specifically. This study has established the general match running activity profiles in elite French female assistant and field referees and notably demonstrates a need to develop their deceleration capacities. It also presents new data on the maximal values attained by officials demonstrating the high physical demands of the game particularly in relation to those observed in players at the very highest standards of play. Finally, the results have demonstrated a trend for a temporal decline in running outputs in both AR and FR. While this latter finding might suggest a need for physical conditioning programmes to counter in-match fatigue, additional research is necessary to explore the possible association between declines in running activity and both contextual factors and individual fitness profiles.
